# Antioxidant Activity Level, Bioactive Compounds, Colour and Spectroscopic Analysis (UV-Vis and FT-IR) of Flavoured Drinks Made with Wine and Sour Cherries (*Prunus* *cerasus* Var. *austera*)

**DOI:** 10.3390/foods10081953

**Published:** 2021-08-22

**Authors:** Michela Pisani, Paola Astolfi, Simona Sabbatini, Patricia Carloni

**Affiliations:** 1 Department of Science and Engineering of Materials, Environment and Urban Planning—SIMAU, Marche Polythecnic University, Via Brecce Bianche 12, I-60131 Ancona, Italy; m.pisani@univpm.it (M.P.); p.astolfi@univpm.it (P.A.); s.sabbatini@univpm.it (S.S.); 2 Department of Agricultural, Food and Environmental Sciences—D3A, Marche Polythecnic University, Via Brecce Bianche, I-60131 Ancona, Italy

**Keywords:** traditional product, ABTS, phenolics, flavonoids, visner, visciole, PCA

## Abstract

In recent years, the increase in consumer interest towards simpler and authentic lifestyles has led to an explosive growth in the production and business of typical agri-food products and, among these, of wines and its derived beverages. With the aim of promoting a typical Italian beverage, the so-called “Vino di visciole” or “Visner”, listed in the national table of traditional agri-food products, the antioxidant and colour properties of fifteen samples from different provinces of the Marche region and obtained with different recipes were analysed. The “in vitro” total antioxidant activity (TAA) determined using ABTS assays, total phenolic content (TPC), total flavonoid content (TFC), total anthocyanins content (TAC), and colour (Somers assay) were measured. In addition, a spectroscopic FT-IR and UV-Vis analysis was carried out to analyse samples with multivariate techniques. The results showed that the production area, the recipe, and the type of cherries used to make the alcoholic beverage do not influence the antioxidant properties and the phytochemical contents of the samples. The multivariate treatment of the spectroscopic features (mainly UV-Vis) rather allowed the differentiation of samples with high antioxidant activity using easy and low-cost instrumental techniques that require little time and can be employed in routine analysis.

## 1. Introduction

One of the predominant trends in agro-industrial markets reveals a growing interest among consumers in traditional products closely linked to a specific place of origin. In fact, customers show a greater propensity to purchase food or agro-industrial products deeply rooted in various popular cultures and perceived as authentic and genuine, even if this means paying higher prices. The growth in international trade, the proliferation of multinational companies with standardised products, and the gradual homogenisation of supplies resulted in an increase in the number of consumers willing to pay a premium to consume regional products that retain the quality of the past and have not been “tainted” by what many people regard as rampant modernisation [1]. Consumers also associate the quality of food with handling along the food production chain and to the correlated chemical risk due to the addition of substances including pesticides, antibiotics, preservatives, and food colouring [2,3].

Nowadays, regional products are not limited to the restricted areas in which they are produced but can reach local, national, or international markets and their “denomination” as regional or typical products is linked to the particularities of their geographic milieu, their quality, and their fame. This identification is a tool for the national and international recognition of the heritage and food culture of the various regions and countries.

Attention to the regionality of foods is often strictly linked to an increased interest in their beneficial effects on human health and on diseases prevention. This results in an enlarged consumer demand for functional foods and, in particular, vegetables and fruits with their phytochemicals that are able to prevent diseases caused by oxidative stress [4]. Phytochemical action has been attributed to their high free radical scavenging capacity that could contribute to, or enhance by induction, the endogenous antioxidant properties of living cells or organisms.

Concerning the antioxidant properties of fruits, a key role is played by grapes and wine as well by berries, cherries, and their juices. In this context, an interesting regional product with high antioxidant potential is represented by a flavoured drink made with wine and sour cherries (*Prunus cerasus* var. *austera*) know by consumers as “Vino di visciole” or “Visner”, and produced in the Marche region (Italy): it is a typical Italian alcoholic flavoured beverage ranging about 14 %vol. Although the recipe followed to produce this beverage can have different variants and can make use of secret natural aromatic additives [5], traditionally, two main production techniques, differing for times and ways of fermentation, can be distinguished and are used in the different zones.

In red grape wine, many phytochemicals are present, providing antioxidant protection with a decrease in platelet aggregation and an increase in endothelial function. Wine polyphenols also prevent and attenuate inflammatory responses through a variety of mechanisms, thereby serving as possible cardioprotective, neuroprotective, and chemopreventive agents [6]. In sour cherries (*Prunus cerasus* L.), phenolic compounds are also present, which have a broad spectrum of beneficial effects for health: for example, they contain significant levels of anthocyanins with strong antioxidant and anti-inflammatory activities [7,8,9,10] and have protective effects on neuronal cells [11]. Moreover, in the past decade, sour cherry products’ utilisation in the food market has increased because of their potential health benefits [12].

Therefore, given the growing consumer interest in traditional products and in their beneficial and genuine aspects, the “in vitro” antioxidant activity and bioactive compounds of different sour cherry beverages, other fruit spirits made with berries from different species of *Prunus* (cherries, black cherries, and blackthorn), and some local red wines were evaluated. To this regard, total antioxidant activity (TAA) using the ABTS assay, total phenolic content (TPC), total flavonoid content (TFC), total anthocyanins content (TAC), and colour were measured [13]. Moreover, in recent years, the use of spectroscopic techniques, such as UV, visible (Vis), near infrared (NIR), and mid-infrared (MIR) integrated with multivariate data analysis, has developed considerably in the de-termination of wine composition (including phenolic compounds). Given their speed and ease of use [14,15], the study of the wine samples was integrated with these analyses and statistically treated with the aim of understanding a possible relationship between composition, production area, and antioxidant properties [16].

## 2. Materials and Methods

### 2.1. Chemicals and Equipment

All reagents, including solvents of the highest purity available, were purchased from Sigma-Aldrich Chemical Co. (Milan, Italy) and used as received. Ultrapure water was used throughout and obtained from a Milli-Q system from Millipore (Milford, MA, USA).

Spectrophotometric measurements were recorded on a Varian Cary 50 UV-Visible spectrophotometer (Agilent Technologies Italia S.p.A., Milano, Italy) or on a microplate reader (Synergy HT, Biotek, Winooski, VT, USA).

### 2.2. Samples

The samples included fifteen (SCW01-15) beverages from wine and sour cherries (*Prunus cerasus* var. *austera*), one (BW) from wine and blackthorn (*Prunus spinosa* L.), one liquor (BCL) made with black cherries (*Prunus cerasus* var. *amarena*) and another liquor (CL) made with cherries (*Prunus avium*); in addition, for comparison, three (W01-03) local red wine were analysed. All samples were purchased from local retail shops. Sour cherry samples were classified according to the area of production declared on the commercial label and are described in detail in Supplementary Materials Table S1, together with all other samples. The production area is important because it is linked to the recipes used in the preparation of the beverage. Usually, in the Ancona province, cherries are initially sun-dried and then macerated with the addition of sugar for the whole summer period (about four months) resulting in a cherry syrup. After harvesting red grape at the end of summer, the must is added to the obtained cherries juice, and the mixture is left to ferment for 2–3 months. After fermentation, the product is filtered, bottled, and seasoned for 2–3 months, typically without the addition of sulphites. Another recipe is commonly adopted in the Pesaro and Urbino province: sugar and red wine (not the red grape must) are added to the fresh cherries in summer, and the resulting mixture is left to ferment for two months; later, alcohol is added and the blend is left to stand for another 5–6 months before bottling. This technique is the most common and used to produce the typical “Visner” [17].

All sample were sub-sampled from freshly opened bottles and stored at −20 °C until analysed.

### 2.3. “In Vitro” Total Antioxidant Activity (TAA) Using ABTS Assay

For measuring the “in vitro” antioxidant activity of the different samples, the ABTS assay was performed according to the method of Pellegrini et al. [18]. The radical cation (ABTS^+•^) was prepared by mixing a 7.0 mM aqueous ABTS (2,2′-azinobis-(3-ethylbenzothiazoline-6-sulfonic acid) diammonium salt) solution with a 2.45 mM aqueous solution of potassium persulfate as oxidising agent in 0.9:0.1 ratio, and allowing the mixture to stand in the dark at room temperature for 12–16 h. Prior to use, the ABTS^+•^ stock solution was diluted ~80:1 with water to obtain an absorbance at 734 nm ranging between 0.6–0.8. To 1.450 mL of this ABTS^+•^ solution, 50 μL of sample previously diluted 100:1 or Trolox (6-hydroxy-2,5,7,8-tetramethylchroman-2-carboxylic acid) standard ethanolic solution appropriately diluted with water, or water as control, were added and mixed. The obtained solution was left for 2 h in the dark at room temperature, and absorbance was read at 734 nm against water. Inhibition percentage values were calculated according to the Equation (1):

Inhibition of A_734_ (%) = (1 − A_c_/A_0_) × 100 (1)

where A_c_ = absorbance of the samples, A_0_ = absorbance of the control.

Antioxidant activity was expressed as mM Trolox Equivalents (TE) using the linear regression value obtained from the Trolox calibration curve.

### 2.4. Total Phenol Content (TPC)

Total phenol content was determined using the Folin–Ciocalteu reagent [19]. To 1.00 mL water, 75 μL of Folin–Ciocalteu reagent followed by 50 μL of sample previously diluted 10:1, or gallic acid standard ethanolic solution appropriately diluted in water, or water as a blank, were added and mixed. After 10 min, 375 μL of 20% Na_2_CO_3_ were added, and the solution was left for 2 h at room temperature in the dark. Absorbance was read at 760 nm, and the results were expressed as mM Gallic Acid Equivalents (GAE) using the linear regression value obtained from the gallic acid calibration curve.

### 2.5. Total Flavonoid Content (TFC)

The total flavonoid content in the samples was measured using a colorimetric assay according to the method of Gursoy et al. [20] with some modifications. Briefly, 50 μL of sample previously diluted 10:1 with water, or (+)-catechin standard ethanolic solution appropriately diluted in water, or water as a blank, were added to 1.350 mL of distilled water. After mixing, 50 μL of 5% NaNO_2_ followed by 50 μL of fresh prepared 10% AlCl_3_ solution in water were added, and the resulting solution was left for 10 min at room temperature in the dark. Absorbance was read at 415 nm, and the results were expressed as mM catechin equivalents (CE) using the linear regression value obtained from the catechin calibration curve.

### 2.6. Total Anthocyanin Content (TAC)

The total monomeric anthocyanin content in the samples was measured by the pH differential method described by Giusti and Wrolstad [21]. Briefly, 100 μL of sample previously diluted 10:1 with water, or water as a blank, were added to 900 μL of 0.025 M potassium chloride buffer, pH 1.0, or 0.4 M sodium acetate buffer, pH 4.5. Absorbances of the two solutions at the different pH were read at 520 and 700 nm. The net absorbance of each sample was calculated as A = (A_520_–A_700_) pH 1.0–(A_520_–A_700_) pH 4.5. The monomeric anthocyanin pigment concentration in the original sample was then calculated as mg/L cyanidine 3-glucoside using the following formula (2):

Monomeric anthocyanin pigment (mg/L) = (A × MW × DF × 1000)/(ε × 1)(2)

where MW is the cyanidine 3-glucoside molecular weight (449.2 g mol^−1^), DF is the dilution factor, and ε is the molar absorptivity of cyanidine 3-glucoside (26,900 L cm^−1^ mol^−1^).

### 2.7. UV-Vis Measurements and Colour Parameters

UV-Vis measurements were performed following the modified Somers assay [22], a set of spectroscopic measurements which can give a measure of wine colour, anthocyanin equilibria, and phenolic composition.

A buffer solution (TB) was prepared dissolving 1.5 g of tartaric acid (0.5% *w*/*v*) in 100 mL of 12% *v*/*v* ethanol solution adjusted to pH 3.4 with 5 M NaOH.

• Treatment “buffer”: 270 μL of the previously prepared TB solution was added to 30 μL of sample and the absorbances at 420 and 520 nm were recorded.
• Treatment “sulphite”: 270 μL of a 0.375% *w*/*v* sodium metabisulfite solution in TB was added to 30 μL of sample and the absorbance at 520 nm was recorded.
• Treatment “acetal”: 270 μL of a 0.1% *v*/*v* acetaldehyde solution in TB was added to 30 μL of sample and after 60 min at room temperature in the dark the absorbances at 420 and 520 nm were recorded.
• Treatment “HCl”: 1470 μL of 1 M HCl solution was added to 30 μL of sample and, after 3 h at room temperature in the dark, the absorbances from 250 to 700 nm were recorded at Δλ = 1 nm constant intervals.
• Colour parameters were calculated as described by Mercurio et al. [22].

### 2.8. FT-IR Instrumentation and Spectra Collection

FT-IR measurements were carried out on all the samples using a Perkin-Elmer FT-IR Spectrometer Spectrum GX1, covering the IR spectral range from 4000 to 950 cm^−1^. A small amount of each sample was dropped onto the centre of the NaCl plates (2 mm thick, 25 mm diameter) and air-dried for 20 min for infrared analysis in transmission mode. Each spectrum was the result of 64 scans with a spectral resolution of 4 cm^−1^. A background spectrum was collected before each acquisition, acquired on clean NaCl salt plate, and ratioed against the sample spectrum. The experiments were carried out at room temperature: i.e., 25 ± 2 °C. For all the samples, the average spectra were calculated (*n* = 5). Raw FT-IR spectra were converted in absorbance and 2-points baseline corrected.

### 2.9. Data Processing

In order to perform an unsupervised analysis, all the FT-IR spectral data in the spectral range 1180–950 cm^−1^ [23,24,25] were corrected according to standard normal variation (SNV), second derivative transformed (DII, 9-point smoothing), mean-centred, and then submitted to Principal Component Analysis. The analysis was used to reduce the entire spectral data set to a small number of latent variables or principal components (PCs) accounting for IR attributes.

An analogous elaboration was performed on UV-Vis spectra recorded from 250 to 700 nm on samples diluted in acidic media (treatment “HCl”) to reduce the entire spectral data set to a small number of latent variables accounting for UV-Vis attributes: in this case, data were only normalised.

The scores of the first three coordinates resulted for each sample from the analysis of FT-IR (PC_FTIR) and UV-Vis (PC_UVVis) spectra were then processed using multivariate chemometric techniques involving cluster analysis (CA) and principal component analysis (PCA).

### 2.10. Statistical Analysis

Appropriate controls were carried out throughout all the experiments described above. The data reported for TPC, TFC, TAC, and TAA represent average values from at least three independent experiments, each performed in duplicate, and the results were expressed as mean with standard deviation (SD). Sour cherry samples were also classified according to the area of production declared on the commercial label (see Supplementary Materials Table S1). Statistical differences were obtained through an analysis of variance (ANOVA) followed by Tukey’s multiple comparison test at 95% confidence level (*p* ≤ 0.05). The normality of the data set was checked with the Jarque–Bera test, and the outliers were determined with the Grubbs test.

UV-Vis spectroscopic measurements were performed in duplicate and averaged at each pin number to produce a single sample spectrum.

Statistical treatments were performed using XLSTAT 2018.5 software (Addinsoft Inc., New York, NY, USA).

Multivariate statistical treatments were performed using Pirouette 4.5 software (InfometrixCorp., Bothell, WA, USA) for FT-IR data and XLSTAT 2018.5 software (Addinsoft Inc., New York, NY, USA) for UV-Vis and PCs data.

## 3. Results

Total antioxidant activity (TAA) using the ABTS assay, total phenolic content (TPC), total flavonoid content (TFC), total anthocyanins content (TAC), and colour parameters, together with spectroscopic data (FT-IR and UV-Vis) of several samples of fruit drinks and red wines, were measured, with the aim of understanding a possible relationship between composition, production area, and antioxidant properties. For sour cherry drinks, particular attention was devoted to any differences between the samples produced with the two different techniques typical of the provinces of Ancona and Pesaro-Urbino.

### 3.1. Total Antioxidant Activity Level, Total Polyphenols, Flavonoids and Anthocyanin Content

Total antioxidant activity using the ABTS assay (TAA), total phenolic content (TPC), total flavonoid content (TFC), and total anthocyanins content (TAC) of all samples are reported in Table 1.

The values obtained from the analysis of the antioxidant activity (TAA) of the samples range from 13 to 30 mM TE for the fruit’s spirits: sour cherry samples SCW15 and SCW01 (13 mM TE) have the lower antioxidant activity values, while SCW06 (28 mM TE) and CL (30 mM TE) have the higher. Values measured for red wines are the highest among all the samples (31–35 mM TE).

**Figure 1:**
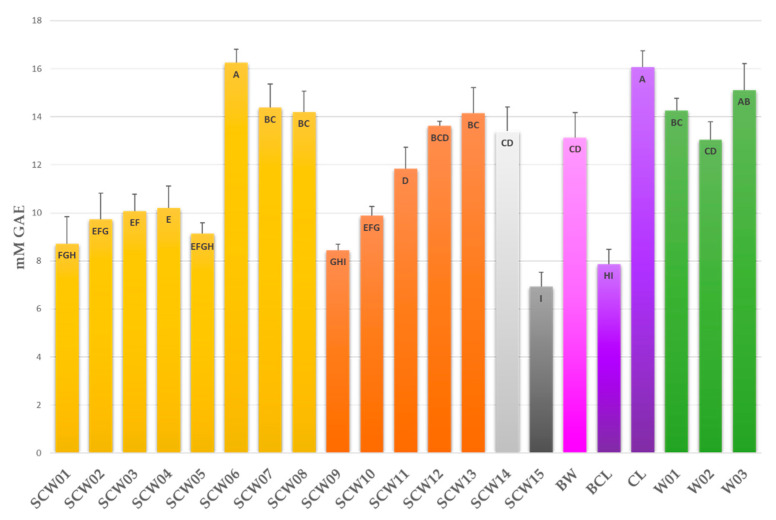
Total phenol contents (TPC) of the samples measured using Folin–Ciocalteu’s reagent. Bars are coloured depending on the province (yellow = PU; orange = AN; light grey = MC; grey = AP) and on the type of the contained berry (yellow, orange, light grey, and grey = sour cherry; pink = blackthorn; violet = cherry; green = grape). Letters in the bars indicate homogeneous sub-classes resulting from Tukey’s post hoc multiple comparison test (*p* < 0.01).

The total phenol contents (TPC) of the samples were measured using Folin–Ciocalteu’s reagent reported in Table 1 are also graphically displayed in Figure 1.

The amount of total phenolics in the samples range between 6.9 and 16.3 mM GAE and agree with data from the literature [23,24,25,26,27]. Sour cherry sample SCW15 (6.9 mM GAE) and black cherry liquor BCL (7.9 mM GAE) contain the lowest amounts of phenolic compounds, while SCW06 (16.3 mM GAE) and CL cherry liquor (16.1 mM GAE) show the highest TPC values.

Samples belonging to the different areas do not show significatively different mean values (AN: 11.6 ± 2.4; PU: 11.6 ± 2.9 mM GAE), while red grape wines show high TPC values (14.1 ± 1.0 mM GAE) comparable to those of the highest sour cherry samples.

Total flavonoids content (TFC) values range from 2.1 to 5.8 mM CE for sour cherry samples and from 5.2 to 5.7 mM CE for red grape wines and agree with the literature reports [23,28,29]. Liquors ranged from 2.7 to 5.8 mM CE.

Total monomeric anthocyanin content (TAC) expressed as mg/L cyanidine-3-glucoside determined with the pH-differential method varies from 12 to 89 mg/L in sour cherry samples. Among these samples, SCW01 contains the lowest amounts of anthocyanins, whereas the highest TAC value was found for SCW13.

The total antioxidant activity trend was compared to data obtained from the quantifications of the main phytochemicals present in the samples showing good and significant correlations between TAA and TPC, TFC and TAC (r = 0.887, 0.849 and 0.815, respectively; *p* < 0.0001), and confirming that all the different classes of phytochemicals contribute to the “in vitro” antioxidant activity. In addition, total flavonoids content (TFC) correlate well with TPC (r = 0.962 and *p* = 0.0000), confirming that flavonoids constitute the most abundant group of polyphenols in red wines [30] and sour cherries [8], but anthocyanins do not correlate with phenols and flavonoids (r = 0.502 and 0.503; *p* = 0.02): in fact, all the red wines show a much higher content of anthocyanins (187 mg/L) than all berry samples, and these values agree with literature reports [31].

### 3.2. Evaluation of Colour Parameters of Samples

Colour parameters for the beverages studied were determined following the modified Somers assay [22] and are reported in Table 2.

Colour density and hue, calculated using the absorbance of the sample at 420 (yellow/orange pigments) and 520 nm (red pigments) in its standardised original state, are routinely used in the wine industry to quantify the visual appearance of wine; strong positive correlations are usually [32] found between colour density and wine quality. Sour cherry samples show colour density values ranging from 5.6 to 14.6 (au) with a significant correlation with TAA, TPC, and TFC (r = 0.482, 0.607 and 0.545, respectively; *p* < 0.05) and hue values ranging from 0.68 and 1.25 that inversely correlate with TAC (r = −0.534; *p* = 0.013).

The two spectral ratios referred to as the “chemical age” express the extent to which polymeric pigment forms displaced the monomers (anthocyanins) during ageing reactions, since the polymeric pigments are artefacts of the wine making process and of subsequent conservation. Chemical Age 1 and 2 increase during ageing and as expected, for sour cherry samples aged at least six months, these parameters are in the ranges from 0.59 to 0.84 and from 0.26 to 0.57, respectively; however, for red wines, lower values (0.4–0.5 and 0.01–0.13, respectively) were obtained. As expected, these parameters inversely correlate with TAC (r = −0.818 and −0.854; *p* < 0.0001).

Analysis of the samples diluted with 1 M hydrochloric acid, which lowers the pH and converts all anthocyanins and many other pigments into their coloured forms, gives an indication of the concentration of total red pigments and total phenolics. These acidified solutions were monitored at 520 and 280 nm, and as expected, it was found that the two measured parameters correlate with TAC (r = 0.993; *p* < 0.0001) and TPC (r = 0.947; *p* < 0.0001), respectively.

### 3.3. Chemometric Analysis of Spectroscopic Data

To investigate the composition of the alcoholic beverages studied, FT-IR and UV-Vis spectroscopic measurements were carried out. The obtained data were statistically processed by principal component analysis (PCA) as multivariate data methods with the Pearson correlation coefficient as the index of similarity between variables.

#### 3.3.1. FT-IR Spectra

Normalised PCA analysis (Figure S1) was performed on the 2nd derivative in the 950–1200 cm^−1^ region of the FT-IR spectra of the samples [15], and results in a model with the first two PCs explain 98% of variation in the spectra (PC1 = 87%, PC2 = 7%, PC3 = 4%). Examination of the PCA score plot of the first two coordinates shows that it is not possible to adequately discriminate between drinks produced from different berries and/or from different areas, and that only red wines separate from the other samples. The eigenvectors of the three first PCs indicates that the bands contributing most to the separation of the samples are those in the range below 1000 and upper 1100 cm^−1^. This model was used to reduce the data dimension and to extrapolate three new variables (PC_FTIR), maximising similarities among samples, and to have a first evaluation of the classificatory efficiency of the variables considered.

#### 3.3.2. UV-VIS Spectra

For UV-Vis spectra, the PCA analysis was performed on the UV-Vis spectra of samples diluted in acid media (Treatment “HCl”) and the resulting first three component explain 96% of variation in the spectra (PC1 = 61%, PC2 = 21%, PC3 = 15%). As can be seen (Supplementary Materials Figure S2) from the score plot (a) and associated eigenvector (b) resulting from the analysis, red wines (green) were sufficiently discriminated from the other samples, but it was not possible to discriminate between SCW produced in different areas, and other fruit samples (CL, BCL, and BW). However, examination of the PCA graph in comparison with antioxidant experimental data reveal that all the SCW samples located on the left have a low antioxidant activity (red ≤ 18 mM TE), whereas the SCW samples located on the right have high TAA values (blue ≥ 21 mM TE) showing that UV-Vis data can help to individuate samples with high antioxidant activity. The eigenvectors of the three first PCs indicates that bands at 270 and 370–590 cm^−1^ (PC1); 255, 300, 370, and 520 cm^−1^ (PC2); and 290–350 cm^−1^ (PC3) are those with the greater variance among the different types of beverages.

This analysis allows us to reduce the UV-Vis data set and to extrapolate three new coordinates (PC_UVVis) maximising similarities among samples and that were then used for whole data set elaboration.

## 4. Discussion

The results obtained in the present study indicate that production area, type of cherries or wine, and procedure used to make the alcoholic beverages have no effect on their antioxidant properties. In fact, sour cherry beverages produced in the PU province show mean values for TAA lower than, but not significantly different from, those of the Ancona area (see Table 1); moreover, the variability of the “in vitro” antioxidant activity between the samples of the two areas is the same. The same trend is also obtained by analysing the TPC and TFC values. On the other hand, the analysis of the results obtained from the TAC measurements permits us to make some consideration of the ageing of the different samples. Red grape wines show a mean value significantly lower than that of PU and AN sour cherry samples (*p* < 0.0001); the mean PU value is lower than that of AN. If the different methods used in the two areas to produce the beverage are considered, it can be stated that the longer ageing of the wine used in the PU province yields a decrease in the anthocyanins content of these beverages. This hypothesis is also supported by the significantly higher anthocyanins content measured for grape red wines that are not aged. In addition, chemical ages (CA) that measure the polymeric pigment formed from monomeric anthocyanins during ageing reactions show an inverse correlation with TAC (r = −0.825 and −0.8541 for CA1 and CA2, respectively; *p* < 0.0001), and monomeric anthocyanins (r = 0.993; *p* < 0.0001), more present in young wines (see Results).

### Statistical Elaboration of Whole Data Set

**Figure 2:**
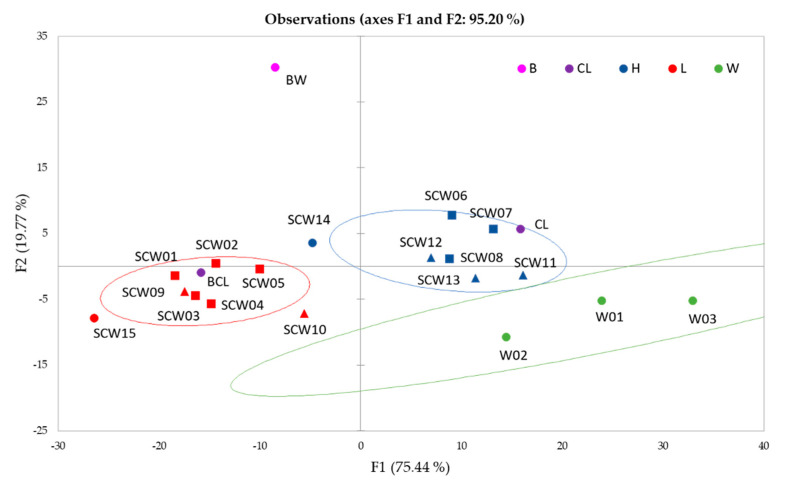
Score plot for the first two factors resulted from the principal component analysis performed using the data set consisting of the first three coordinates (PC_FTIR) obtained from the analysis of the FT-IR spectra and of the UV-Vis spectra in HCl solutions. Points are coloured depending on the type of the contained berry and on the TAA level: (red = sour cherry with low TAA; blue = sour cherry with high TAA; pink = blackthorn; violet = cherry liquors; green = grape wine) and shaped depending on the production area (square = PU; triangle = AN).

Although PCA itself cannot be used as a classification tool, it can indicate a trend that is relevant for visualising the dimension of the space. With this aim, the first three coordinates obtained from the PCA analysis of the FT-IR spectra (PC_FTIR) and the first three coordinates obtained from the PCA analysis of the UV-Vis spectra in HCl solutions (PC_UVVis), were statistically elaborated by performing a principal component analysis using the covariance as the index of similarity between variables. The score plot resulting from the analysis and displayed in Figure 2 shows that the first two factors (F) explain 95.2% of variation in the spectra (F1 = 75.4%, F2 = 19.8%) and discriminate well between W, SCW with high (H) and low (L) antioxidant activity, and BW (B), whereas liquors (CL) are arranged following their antioxidant activity rather than the type of cherry.

The eigenvectors of the first three factors reported in Table 3 show that the FT-IR coordinates contribute very little to the separation of the samples, and it is especially UV-Vis variables that give an important contribution to the determination of the antioxidant potential of the samples.

In addition, a cluster analysis (CA) was applied to determine whether the data set could be divided into groups to explain the scale and relationship of the different type of samples analysed. The similarities between the samples were determined based on their Euclidean distance, and the objects were clustered using Ward’s method.

**Figure 3:**
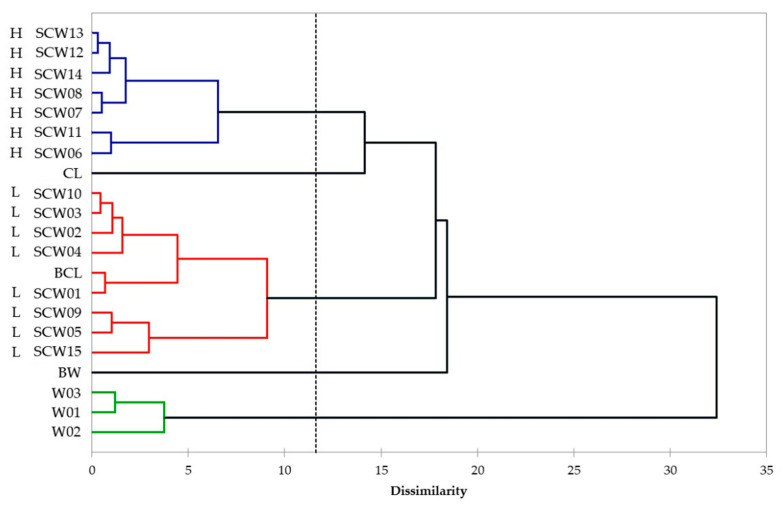
Dendrogram obtained by cluster analysis performed using Ward’s method (Euclidean distance) on the data set incorporating all samples and six variables (PCs_FTIR and PCs_UVVis). SCW samples are described by their acronym and their antioxidant activity level as L and H. Groups are coloured depending on the type of beverage and on the TAA level: (red = sour cherry with low TAA; blue = sour cherry with high TAA; green = grape wine).

The CA dendrogram displayed in Figure 3 shows that three main clusters were suggested. The first group consisted of SCW samples with high antioxidant activity (H), the second group of SCW with low antioxidant activity (L), and the third consisted of red wines. In addition, one of the CL sample and BW sample were separated from all other beverages.

## 5. Conclusions

In this paper, several samples of sour cherry spirits produced in the Marche region with different typical recipes were studied to evidence the beneficial properties of these beverages and to correlate this feature to the different techniques utilised for the production.

The results showed that the production area, the recipe, and the type of cherries used to make the alcoholic beverage do not influence the antioxidant properties and the phytochemical contents of the samples.

Moreover, high “in vitro” antioxidant activity values were measured for sour cherry beverages that can be ascribed more to flavonoids than to anthocyanin’s content.

In addition, the multivariate treatment allowed us to differentiate samples with high antioxidant activity using easy and low-cost instrumental techniques that require little time and can be employed in routine analysis.

In conclusion, the characterisation of antioxidant activity and colour of this typical beverage widespread in the Marche region could be used to promote the production and the commercialisation of this traditional product.

## Figures and Tables

**Table 1:** Total antioxidant activity using the ABTS assay (TAA), total phenolic content (TPC), total flavonoid content (TFC), and total anthocyanins content (TAC) of all samples.

Sample	TAA	TPC	TFC	TAC
	mM TE	mM GAE	mM CE	mg/L
SCW01	13 ± 4	8.7 ± 1.1	3.4 ± 0.3	12 ± 3
SCW02	16 ± 2	9.7 ± 1.1	3.5 ± 0.2	24 ± 7
SCW03	17 ± 2	10.1 ± 0.7	3.2 ± 0.2	41 ± 7
SCW04	18 ± 3	10.2 ± 0.9	3.2 ± 0.2	52 ± 8
SCW05	18 ± 1	9.1 ± 0.4	3.5 ± 0.4	31 ± 5
SCW06	28 ± 2	16.3 ± 0.6	5.7 ± 0.3	41 ± 6
SCW07	21 ± 2	14.4 ± 1.0	5.9 ± 0.4	41 ± 4
SCW08	25 ± 1	14.2 ± 0.9	5.4 ± 0.3	39 ± 7
SCW09	14 ± 2	8.4 ± 0.2	3.0 ± 0.2	22 ± 4
SCW10	18 ± 3	9.9 ± 0.4	3.1 ± 0.2	51 ± 8
SCW11	22 ± 3	11.8 ± 0.9	4.3 ± 0.2	44 ± 3
SCW12	25 ± 2	13.6 ± 0.2	4.7 ± 0.3	67 ± 4
SCW13	27 ± 1	14.2 ± 1.1	4.9 ± 0.2	89 ± 4
SCW14	24 ± 4	13.4 ± 1.0	4.8 ± 0.2	26 ± 5
SCW15	13 ± 2	6.9 ± 0.6	2.1 ± 0.1	18 ± 2
BW	20 ± 2	13.1 ± 1.0	5.5 ± 0.5	18 ± 3
BCL	14 ± 2	7.9 ± 0.6	2.8 ± 0.2	16 ± 4
CL	30 ± 2	16.1 ± 0.7	5.9 ± 0.6	72 ± 4
W01	31 ± 1	14.3 ± 0.5	5.6 ± 0.3	174 ± 6
W02	31 ± 2	13.1 ± 0.7	5.2 ± 0.3	178 ± 6
W03	35 ± 3	15.1 ± 1.1	5.7 ± 0.3	208 ± 8
SCW(PU)	19 ± 5 ^B^	11.6 ± 2.9 ^A^	4.2 ± 1.2 ^A^	35 ± 12 ^B^
SCW(AN)	21 ± 5 ^B^	11.6 ± 2.4 ^A^	4.0 ± 0.9 ^A^	55 ± 25 ^B^
W	32 ± 2 ^A^	14.1 ± 1.0 ^A^	5.5 ± 0.3 ^A^	187 ± 18 ^A^

Results are expressed as mean values ± standard deviation (*n* = 3). Superscript letters within each column indicate homogeneous sub-classes resulting from Tukey’s post hoc multiple comparison test (*p* < 0.01).

**Table 2:** Colour parameter calculated for all samples.

Sample	Area	Colour Density	Hue	Chemical Age 1	Chemical Age 2	Total Anthocyanins	Total Phenolics
SCW01	PU	7.9	1.25	0.84	0.53	24.8	46.3
SCW02	PU	8.3	1.02	0.82	0.51	32.4	48.1
SCW03	PU	7.2	0.80	0.63	0.31	89.6	43.7
SCW04	PU	8.8	0.76	0.59	0.27	134.4	47.8
SCW05	PU	8.8	0.89	0.71	0.41	81.1	54.4
SCW06	PU	13.3	0.99	0.80	0.44	86.8	77.7
SCW07	PU	14.4	0.80	0.79	0.44	108.1	79.8
SCW08	PU	13.9	0.68	0.78	0.44	105.6	69.1
SCW09	AN	6.9	0.87	0.71	0.35	76.5	45.1
SCW10	AN	10.6	0.75	0.66	0.32	133.3	43.7
SCW11	AN	14.6	0.77	0.76	0.40	130.7	61.5
SCW12	AN	10.8	0.89	0.66	0.26	191.1	64.6
SCW13	AN	12.3	0.68	0.68	0.27	228.9	69.9
SCW14	MC	9.2	0.83	0.83	0.43	73.1	67.8
SCW15	AP	5.6	0.97	0.64	0.32	63.1	31.5
BW		4.9	1.09	0.71	0.33	53.5	73.4
BCL		10.4	1.14	0.70	0.57	23.1	47.0
CL		13.0	0.73	0.69	0.33	165.8	80.3
W01		11.2	0.79	0.51	0.13	409.4	76.5
W02		8.3	0.70	0.47	0.09	448.5	67.0
W03		10.3	0.74	0.44	0.09	559.6	85.5

**Table 3:** Eigenvalues, explained (Variability %) and cumulative variance (Cumulative %), and eigenvectors for the first three factors (F) obtained with principal component analysis performed on the data set consisting of the first three coordinates (PC_FTIR) obtained from the FT-IR spectra and the first three coordinates (PC_UVVis) obtained from the UV-Vis spectra in HCl solutions.

	F1	F2	F3
Eigenvalue	257.119	67.382	16.325
Variability (%)	75.436	19.769	4.790
Cumulative %	75.436	95.205	99.994
Eigenvectors			
PC_FTIR1	0.005	−0.009	−0.024
PC_FTIR2	0.000	0.000	0.000
PC_FTIR3	0.001	−0.001	−0.003
PC_UVVis1	1.000	0.000	0.000
PC_UVVis2	0.000	1.000	0.000
PC_UVVis3	0.000	0.000	1.000

## Data Availability

Data is contained within the article or Supplementary Materials.

## Supplementary Materials

The following are available online at https://www.mdpi.com/article/10.3390/foods10081953/s1. Table S1. Description of the samples. Figure S1. Score plot (a) for the first two PCs, eigenvector (b) and eigenvalue (c) resulted from the PCA analysis performed on the 2nd derivative of the 950–1180 cm^−1^ region of the FT-IR spectra of the samples. Figure S2. Score plot (a) for the first two PCs, eigenvector (b) and eigenvalue (c) resulted from the PCA analysis performed on the UV-Vis spectra of samples diluted in acid media (Treatment “HCl”).
