# Supplementary material for: Antioxidant Activity Level, Bioactive Compounds, Colour and Spectroscopic Analysis (UV-Vis and FT-IR) of Flavoured Drinks Made with Wine and Sour Cherries (Prunus cerasus Var. austera)
Source: Foods. 2021 Aug 22;10(8):1953. doi: 10.3390/foods10081953 (PMC8393568; doi:10.3390/foods10081953)
Supplement: Supplementary file 1 [file foods-10-01953-s001.zip › Figure S1.pdf]

Figure S1. Score plot (a) for the first two PCs, eigenvector (b) and eigenvalue (c) resulted from the PCA analysis performed on the 2nd derivative of the 950-1180 cm^{-1} region of the FTIR spectra of the samples. Points are coloured depending on the province (yellow = PU; orange = AN; light grey = MC; grey = AP) and on the type of the contained berry (yellow, orange, light grey and grey = sour cherry; pink = blackthorn; violet = cherry; green = grape).

a)

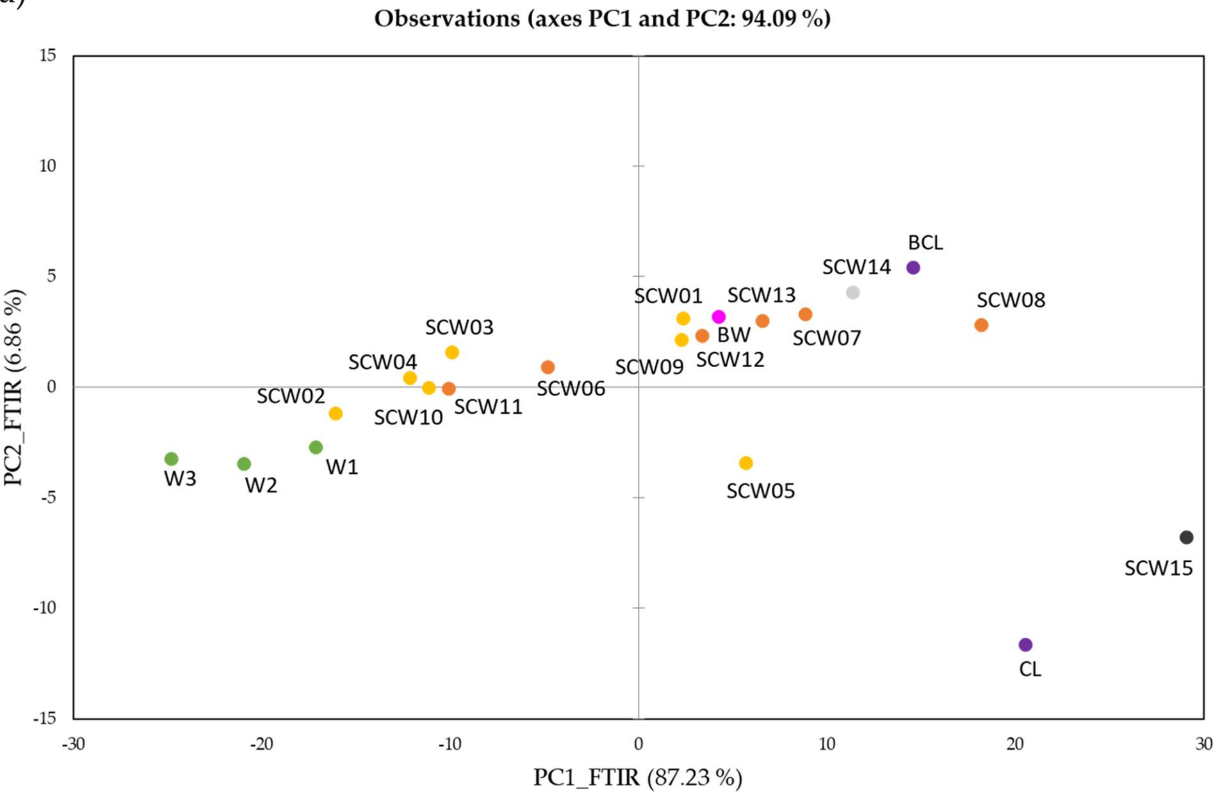

b)

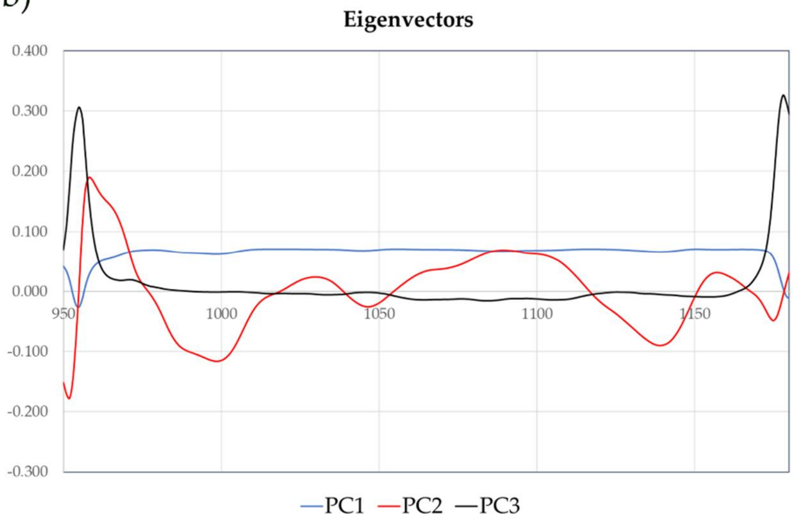

c)

	F1	F2	F3
Eigenvalue	201.499	15.848	8.368
Variability (%)	87.229	6.861	3.622
Cumulative %	87.229	94.090	97.712
