# Supplementary material for: Antioxidant Activity Level, Bioactive Compounds, Colour and Spectroscopic Analysis (UV-Vis and FT-IR) of Flavoured Drinks Made with Wine and Sour Cherries (Prunus cerasus Var. austera)
Source: Foods. 2021 Aug 22;10(8):1953. doi: 10.3390/foods10081953 (PMC8393568; doi:10.3390/foods10081953)
Supplement: Supplementary file 1 [file foods-10-01953-s001.zip › Figure S2.pdf]

Figure S2. Score plot (a) for the first two PCs, eigenvector (b) and eigenvalue (c) resulted from the PCA analysis performed on the UV-Vis spectra of samples diluted in acid media (Treatment “HCl”). Points are coloured depending on the province (yellow = PU; orange = AN; light grey = MC; grey = AP) and on the type of the contained berry (yellow, orange, light grey and grey = sour cherry; pink = blackthorn; violet = cherry; green = grape). Circles group SCW samples with low (red; TAA ≤ 18 mM TE) or high (blue; TAA ≥ 21 mM TE) antioxidant activity.

a)

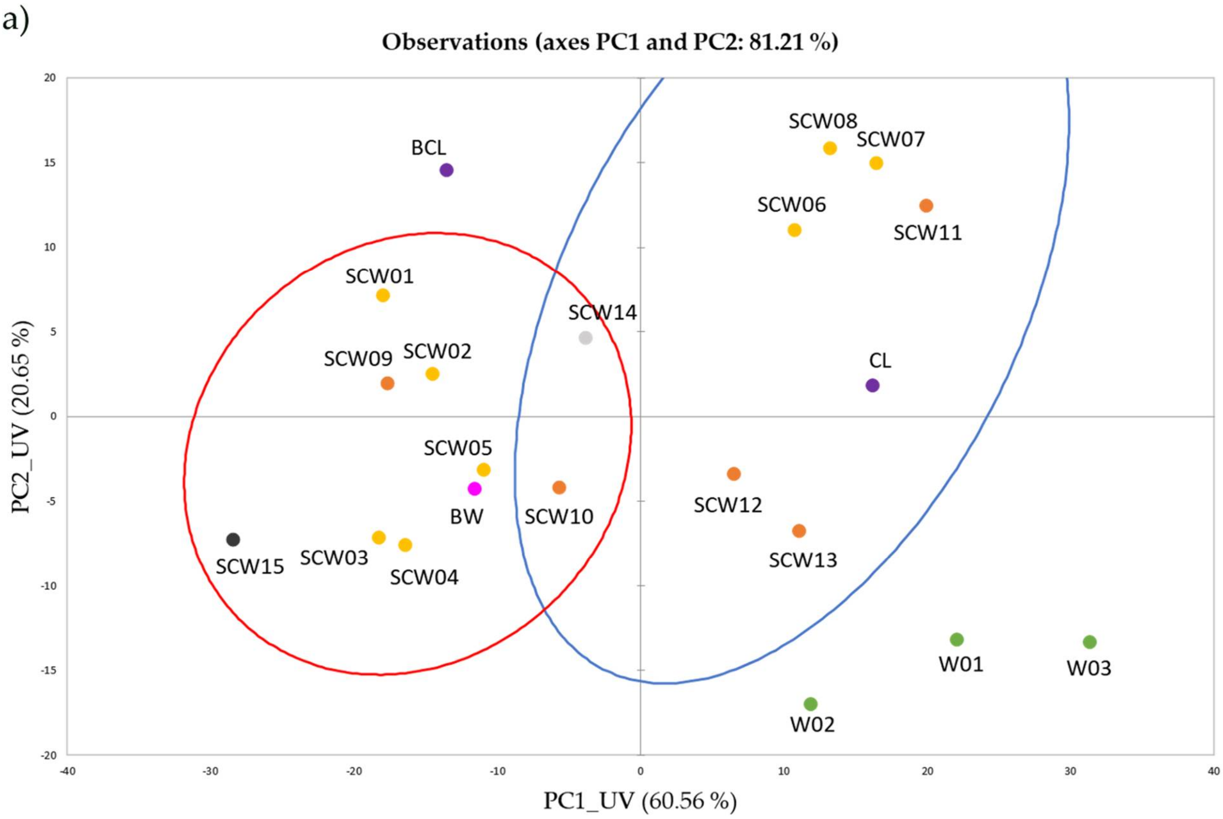

b)

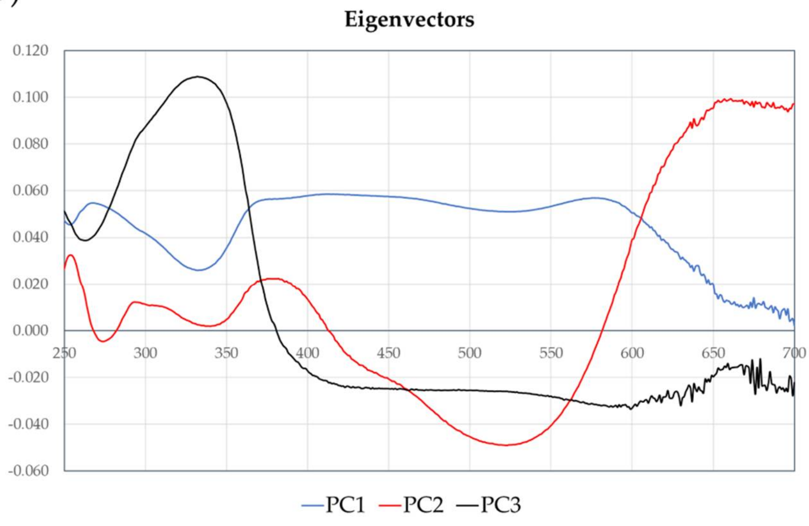

c)

	PC1	PC2	PC3
Eigenvalue	273.119	93.134	66.015
Variability (%)	60.559	20.650	14.638
Cumulative %	60.559	81.209	95.847
