# Supplementary material for: Antioxidant Activity Level, Bioactive Compounds, Colour and Spectroscopic Analysis (UV-Vis and FT-IR) of Flavoured Drinks Made with Wine and Sour Cherries (Prunus cerasus Var. austera)
Source: Foods. 2021 Aug 22;10(8):1953. doi: 10.3390/foods10081953 (PMC8393568; doi:10.3390/foods10081953)
Supplement: Supplementary file 1 [file foods-10-01953-s001.zip › Table S1.pdf]

Table S1. Description of the origin of the samples.

Code	Commercial Name	Berries	Location	Province	
SCW01	Visciolì	Sour cherry	Pergola	Pesaro-Urbino	PU
SCW02	Visciolata	Sour cherry	Acqualagna	Pesaro-Urbino	PU
SCW03	Vino & Visciole	Sour cherry	Cantiano	Pesaro-Urbino	PU
SCW04	Visner 2010	Sour cherry	Pergola	Pesaro-Urbino	PU
SCW05	Visner Selezione 2010	Sour cherry	Pergola	Pesaro-Urbino	PU
SCW06	Visner Selezione 2008	Sour cherry	Pergola	Pesaro-Urbino	PU
SCW07	Visciola	Sour cherry	Isola del Piano	Pesaro-Urbino	PU
SCW08	Cerasus di Cantiano 2010	Sour cherry	Cantiano	Pesaro-Urbino	PU
SCW09	Centounopercento	Sour cherry	Cupramontana	Ancona	AN
SCW10	L'Orcio	Sour cherry	San Marcello	Ancona	AN
SCW11	Sicomoro	Sour cherry	San Marcello	Ancona	AN
SCW12	Visciole Velenosi	Sour cherry	San Marcello	Ancona	AN
SCW13	Vino di Visc. Pozzo Buono	Sour cherry	Morro D'Alba	Ancona	AN
SCW14	Bacco non lo sa	Sour cherry	Macerata	Macerata	MC
SCW15	Vino di visciole	Sour cherry	Stella di Monsanpolo	Ascoli Piceno	AP
BW	Prunus	Blackthorn	Pergola	Pesaro-Urbino	PU
BCL	Ratafià	Black cherry	S. Giovanni Teatino	Chieti	CH
CL	Ratafià	Cherry	Tocco da Casauria	Pesacara	PE
W01	Arciere (Lacrima)	Grape	San Marcello	Ancona	AN
W02	Ancillotto (Lacrima)	Grape	San Marcello	Ancona	AN
W03	Giocoliere (Sangiovese)	Grape	San Marcello	Ancona	AN
